# Density-dependent immune responses against the gastrointestinal nematode *Strongyloides ratti*

**DOI:** 10.1016/j.ijpara.2007.04.023

**Published:** 2007-11

**Authors:** Colin Bleay, Clare P. Wilkes, Steve Paterson, Mark E. Viney

**Affiliations:** a School of Biological Sciences, University of Bristol, Woodland Road, Bristol, BS8 1UG, UK; b School of Biological Sciences, University of Liverpool, Liverpool, L69 7ZB, UK

**Keywords:** Density-dependent selection, Immune response, Helminth, Survivorship, Fecundity

## Abstract

Negative density-dependent effects on the fitness of parasite populations are an important force in their population dynamics. For the parasitic nematode *Strongyloides ratti*, density-dependent fitness effects require the rat host immune response. By analysis of both measurements of components of parasite fitness and of the host immune response to different doses of *S. ratti* infection, we have identified specific parts of the host immune response underlying the negative density-dependent effects on the fitness of *S. ratti*. The host immune response changes both qualitatively from an inflammatory Th1- to a Th2-type immune profile and the Th2-type response increases quantitatively, as the density of *S. ratti* infection increases. Parasite survivorship was significantly negatively related to the concentration of parasite-specific IgG_1_ and IgA, whereas parasite fecundity was significantly negatively related to the concentration of IgA only.

## 1 Introduction

Negative density-dependent effects on components of fitness act on virtually all organisms, which ultimately have powerful effects on population dynamics. How these density-dependent effects occur will also affect the population-level consequences, including how the system will respond to natural or artificial perturbation. For all organisms (parasites and free-living), density-dependent effects may occur via crowding, in which competition for limiting resources (e.g., nutrients, space) is increased in high-density settings with negative consequences for fitness. However, for parasites in which the environment is a host, density-dependent effects may also be mediated by the host immune response (Anderson and Michel, 1977; Paterson and Viney, 2002).

For helminth parasites, including nematodes, density-dependent effects are most often seen as a reduction in fecundity and parasitic adult survivorship with an increase in intensity of infection (Dezfuli et al., 2002; Keymer, 1982; Lowrie et al., 2004; Quinnell et al., 1990). For the gastrointestinal parasitic nematode of rats, *Strongyloides ratti*, density-dependent effects on the survivorship and fecundity of the parasitic adult stage are dependent on the host immune response (Paterson and Viney, 2002). The evidence for this is that these density-dependent effects only occur late in a primary infection (i.e., as an anti-*S. ratti* immune response develops) and that these effects do not occur in hosts that are unable to mount an effective immune response (Paterson and Viney, 2002). However, it is not known what components of the host anti-*S. ratti* immune response underlie these effects. Therefore, the purpose of this study was: (i) to determine how the host immune response to an *S. ratti* infection changes both quantitatively and/or qualitatively with variation in parasite density; (ii) to identify the putative effectors of the immune-dependent, density-dependent reduction in the fitness of *S. ratti.*

Analysis of the vertebrate immune response to a range of helminth species has shown that the response is typically a T-helper type 2 (Th2) response, rather than an inflammatory Th1-type response that is generated in response to microparasites (Finkelman et al., 1997; Grencis et al., 1991; Kringel et al., 2006). A Th2-type immune response is characterised by the production of the cytokines IL-4, IL-5, IL-13 and IgA, IgE and, in rodents, IgG_1_ antibody responses (Jankovic et al., 2006). The reasons why such Th2-type responses are so characteristic of helminth infections are not clear, but are likely to be multifaceted. Helminth antigens, particularly the glycan components, appear to have some inherent ability to elicit a Th2-type response (Tawill et al., 2004). Despite the commonality of the type of immune response of mammalian hosts to helminth infections, the putative effectors of reductions in helminth fitness, including clearance from the host, differ between species and in most cases proof of the role of these effectors is still wanting (Artis and Grencis, 2001).

*Strongyloides ratti* is a gastrointestinal nematode parasite of the rat. Infections in rats are typically acute, being completely cleared after approximately 30 days (Carter and Wilson, 1989), though low level, longer lived infections also occur (Kimura et al., 1999). Hosts become infected when infective L3s (iL3s) penetrate the skin of their host and migrate via the cranium and nasal–frontal region (Koga et al., 1999; Tindall and Wilson, 1988) to the gut, where they moult via an L4 stage into adult females only, which reproduce by parthenogenesis (Viney, 1994). These adult stages occupy the proximal ∼40% of the small intestine and lie in its mucosa, usually close to the crypts of Lieberkühn (Dawkins et al., 1983). This process of maturation and migration takes approximately 3 days with parasitic females achieving maximal size 3 days p.i. (Viney et al., 2006). As the host immune response develops, *S. ratti* parasitic females become shorter, their fecundity is reduced and they move to more posterior positions in the small intestine (Kimura et al., 1999; Wilkes et al., 2004). However, these effects are reversible, such that if worms are surgically transplanted to naïve hosts or if the host is immunosuppressed, then the parasitic female worms regain their size and fecundity (Moqbel et al., 1980; Viney et al., 2006).

There has been immunological analysis particularly of both *S. ratti* and *Strongyloides stercoralis* in natural rat and dog (or human) hosts, respectively, as well as non-natural, but convenient, laboratory hosts. The available data are broadly consistent with the development of a Th2-type immune response, in common with other nematodes, and with the induction of a substantial intestinal mast cell response as part of the anti-*Strongyloides* effector mechanism (Abe et al., 1993; Artis and Grencis, 2001; Miller, 1984). The transfer of serum from *S. ratti*-infected to -naïve rats, transfers resistance to an *S. ratti* infection, with this effect concentrated in the IgG_1_ fraction (Murrell, 1981). This is consistent with the observed temporal change in anti-*S. ratti* IgG_1_ responses in *S. ratti*-infected rats, though a similar change in anti-*S. ratti* IgG_2a_ was also seen (Wilkes et al., 2007). In addition, total serum IgE, intestinal anti-*S. ratti* IgA and rat mast cell protease II (RMCP II), and the concentration of IL-4 produced by mesenteric lymph node (MLN) cells in response to stimulation with parasitic female antigen all increase in response to *S. ratti* infection (Wilkes et al., 2007). Analysis of repeated different doses of *S. ratti* infection in rats have shown that there is a dose-dependent anti-*S. ratti* IgG and IgE response (Uchikawa et al., 1991). However no attempt has been made to relate anti-*S. ratti* immune responses to negative density-dependent effects on the fitness of *S. ratti*.

There has been limited analysis of the effect of helminth dose on the host immune response. For the gastrointestinal nematode *Trichuris muris*, there is a Th1 cytokine profile for low dose infections, but a Th2-type cytokine profile for high dose infections; parasite survivorship was also greater in the low dose infections (Bancroft et al., 1994). For the tissue dwelling filarial nematode *Litomosoides sigmodontis*, there are negative density-dependent effects on survivorship and female reproductive status and effects of parasite dose on the magnitude of the host immune response with some suggestion of greater polarisation to a Th2-type response at high parasite doses (Babayan et al., 2005). For the tapeworm *Echinococcus granulosus* there is a mixed Th1- and Th2-type cytokine profile (a so-called Th0 response) in low dose infections, but a Th2 response for high dose infections. However, there did not appear to be negative density-dependent effects on the *E. granulosus* stages in the host (Dematteis et al., 2003).

In summary, parasitic nematodes are subject to negative density-dependent effects that for *S. ratti* are immune-dependent. Parasitic nematodes generate a Th2-type immune response, the magnitude of which may be related to parasite dose. Here, we have determined the quantitative and qualitative change in the host immune response to different doses of *S. ratti* infection, to thereby seek to understand what components of the host immune response are associated with the density-dependent reduction in the survivorship and fecundity of *S. ratti*.

## 2 Materials and methods

### 2.1 Parasites

The *S. ratti* isofemale line ED321 Heterogonic (Viney, 1996) was used throughout. Forty-five female Wistar rats of approximately 100 g were allocated equally to one of five dose treatments and were administered s.c. with 0, 6, 30, 150 or 750 iL3s on day 0 p.i.; animals receiving 0 worms were administered with an equal volume of PBS (Wilkes et al., 2004). This range of infective doses encompass that found in *S. ratti* infections of wild rats (Fisher and Viney, 1998). This experiment was conducted in three equal experimental blocks of 15 animals, with each block separated by 1 day. Within each experimental block, faecal samples were collected from one rat from each dose treatment group on days 7, 14 or 21 p.i. and cultured (Viney, 1996). The number of larvae that developed in these cultures was used as a measure of the total viable egg output of the infection (Gemmill et al., 1997). These same animals were then sacrificed on days 8, 15 or 22 p.i., respectively, both to determine the number of parasitic females present in the gut and to collect tissue and serum samples for immunological assays. These sampling points encompassed the previously observed temporal changes in rat anti-*S. ratti* immune response (Wilkes et al., 2007). The small intestine was also collected by dissection and stored at −20 °C prior to processing. The number of parasitic females within the anterior 10%, by length, of the small intestine was determined by microscopical examination (Wilkes et al., 2004). Because the relative position of parasitic female worms in the host intestine becomes more posterior as a host immune response develops (Kimura et al., 1999; Wilkes et al., 2004), determination of the number of females in the anterior-most 10% of the gut is likely to result in an underestimate of the number of parasitic females, particularly later in infection, such that their survivorship and per capita fecundity may be under- and over-estimated, respectively.

These experiments were conducted under licences issued with the authority of the UK Animals (Scientific Procedures) Act 1986.

### 2.2 Serum and tissue samples

Blood samples were taken immediately after sacrifice by cardiac puncture; the spleen and MLN were recovered by dissection as previously described (Wilkes et al., 2007). To sample small intestinal tissue for immunological analysis, 1 g of tissue posterior to the anterior 10% used for determination of the number of parasitic females (above), was isolated and prepared as previously described (Wilkes et al., 2007).

### 2.3 Immunological assays

The components of the rats’ immune response that were measured were as follows: IL-4, IL-13 and IFN-γ from both the spleen and MLN cells in response to stimulation with parasitic female antigen and ConA; parasite-specific IgG_1_, IgG_2a_ and IgG_2b_ in serum; total IgE in serum and parasite specific IgA and RMCP II in intestinal tissue. All of these assays were performed as previously described (Wilkes et al., 2007) except that the measure of IgG_1_ was made with respect to recombinant IgG_1_ (Serotec, UK) as described for IgG_2a_ and IgG_2b_ (Wilkes et al., 2007). IL-13 was assayed in supernatants of stimulated MLN and spleen cells using commercially available sandwich ELISA kits (Biosource International Inc., USA), following the manufacturer’s instructions.

### 2.4 Statistical analysis

The effect of infective ln Dose and Time p.i. on measures of components of the host immune response were analysed using a tobit regression model (Tobin, 1958). This was done because ELISA absorbance readings that fell below the lower limit of the standard curve were not detectable and were therefore set to zero leading to an inflation of the zero class in the dataset, i.e., the data were left censored. The procedure followed is outlined elsewhere (Han and Kronmal, 2004). Briefly, data were first normalised using an iterative Box-Cox transformation to determine the value of *λ* that was used in the transformation, which maximised the log-likelihood of the tobit model (Han and Kronmal, 2004). The tobit regression model assumes that above a threshold for censored data, the data are independent and normally distributed. Bonferroni corrections were applied in analyses of fixed effects on ln Dose and Time p.i. to achieve a corrected alpha level equivalent to 0.0027.

Generalised linear models (GLM) with negative binomial error structures were used to investigate: (i) the relationship between the fixed effects of infective ln Dose and Time p.i. and the survivorship and fecundity of *S. ratti*; and (ii) the relationship between measured immune parameters (identified as being significantly affected by ln Dose and, or Time p.i using the tobit regression analysis (above)), and the survivorship and fecundity of *S. ratti*.

In the GLM analysis of survivorship and fecundity the offset variables of ln Dose (the number of iL3s administered) and ln Adults (the number of parasitic females present in the gut at time of sample), respectively, were used. The effect of this in these models was that the response variable and offset variable were the numerator and denominator, respectively. Thus, in this way, survivorship is the probability that an administered iL3s survives as an adult parasite until the time of sampling; fecundity is the average number of viable eggs produced by adult parasitic females present at the time of sampling (Paterson and Viney, 2002).

Quadratic terms were included where the relationship between explanatory variables and response variables appeared to be curvilinear. The significance of an explanatory variable in a model was determined by deletion testing, where the significance of any variable was established with a log-likelihood ratio test (Paterson and Viney, 2002). All analyses were carried out using the statistical software R (v2.3.1) (http://www.r-project.org).

## 3 Results

### 3.1 Density-dependent effects on *S. ratti* fitness

There were negative density-dependent effects on parasite survivorship and fecundity. There were significant effects of Time p.i., and of an interaction between Time and ln Dose, on the survivorship of *S. ratti*; there was no significant effect of ln Dose (Table 1). Thus, there was a greater negative density-dependent effect on survivorship as an infection progresses, and this was greater still in infections initiated with high infective doses. This is consistent with previous observations of density-dependent effects in *S. ratti* (Paterson and Viney, 2002).

There were significant effects of Time p.i. and of ln Dose on parasite fecundity (Table 1). However, the direction of these density-dependent effects was different, negative for Time p.i. (consistent with the effect on parasite survivorship) but positive for Dose (Table 1). This negative effect of time p.i. has been observed previously (Paterson and Viney, 2002). In this same previous study, there was evidence of a positive density-dependent effect of infective dose on fecundity early in infection, which became a negative effect later in infection (Paterson and Viney, 2002).

### 3.2 *Strongyloides ratti* infection and the host immune response

**Fig. 1:**
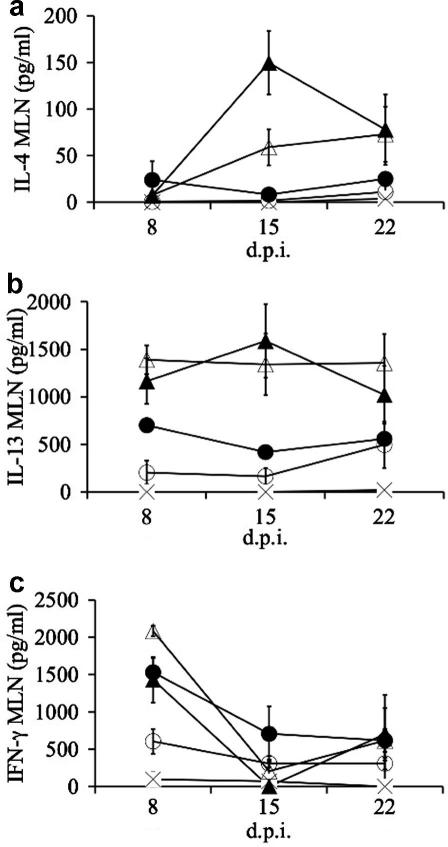
The mean concentration of (a) IL-4, (b) IL-13 and (c) IFN-γ by mesenteric lymph node (MLN) cells stimulated with parasitic female antigen from animals infected with different doses of *Strongyloides ratti*, where x = 0, ○ = 6, • = 30, ▵ = 150, ▴ = 750 infective larvae at different days p.i. (d.p.i.). Error bars are ±1 SEM.

**Fig. 2:**
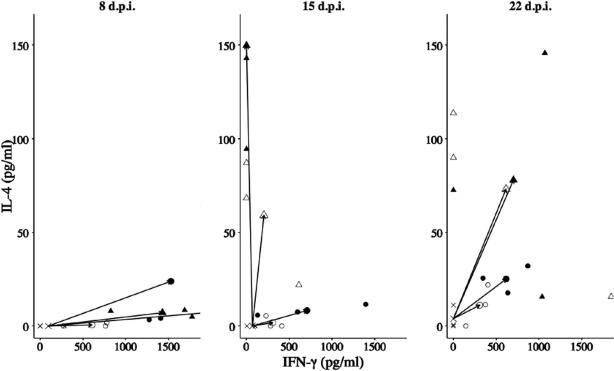
Vectors calculated as the mean within-infective dose concentration of IFN-γ and IL-4 of mesenteric lymph node (MLN) cells stimulated with parasitic female antigen, compared with the mean values of the control animals (dose = 0) at 8, 15 and 22 days p.i. (d.p.i.), where x = 0, ○ = 6, • = 30, ▵ = 150, ▴ = 750 infective larvae. Large symbols are the means for each dose treatment; small symbols are individual data points.

There was a positive *S. ratti* density-dependent effect on the concentration of IgA, IgG_1_, RMCP II and IL-4, and IL-13 produced by MLN cells. Overall, this suggests that infection with *S. ratti* induces a Th2-type response, the size of which was determined by the size of the infective dose. For the MLN cells stimulated with parasitic female antigen, the concentration of IL-4 and IL-13 was significantly positively affected by ln Dose; there was no such effect on the concentration of IFN-γ (Fig. 1). Production of IL-4 and IL-13 is representative of a Th2-type cytokine profile. The concentration of IL-4, IL-13 and IFN-γ produced by spleen cells were lower than that produced by MLN cells in response to stimulation with parasitic female antigen (data not shown). There were no significant effects of *S. ratti* infective dose on the concentration of IL-4, IL-13 or IFN-γ produced by spleen cells when stimulated with parasitic female antigen (data not shown). Using the combination of the concentration of IFN-γ and IL-4 produced by MLN cells stimulated with parasitic female antigen as a measure of a Th1- and Th2-type response, respectively, this shows that rats infected with *S. ratti* alter their immune response from a Th1- to a Th2-type response as an infection progresses, with this change greater in animals infected with the highest doses (Fig. 2). Thus, at 8 days p.i. there was a Th1-type profile for all infective doses; however, at days 15 and 22 p.i. this became a Th2-type profile, especially for the two highest infective doses (150 and 750 infective larvae); the lowest infective doses (six and 30 infective larvae) had a mixed Th1- and Th2-type profile at both 15 and 22 days p.i. (Fig. 2). This is therefore consistent with a *S. ratti*-dose and time p.i.-dependent development of a Th2-type cytokine profile.

**Fig. 3:**
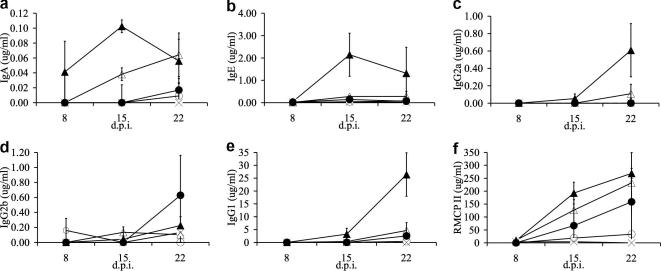
The mean concentration of (a) parasite-specific IgA in intestinal tissue; (b) total serum IgE; parasite-specific serum; (c) IgG_2a_; (d) IgG_2b_; (e) IgG_1_ and (f) RMCP II in intestinal tissue from animals infected with different doses of *Strongyloides ratti*, where x = 0, ○ = 6, • = 30, ▵ = 150, ▴ = 750 infective larvae at different days p.i. (d.p.i.). Error bars are ±1 SEM.

There were significant positive effects of both Time p.i. and ln Dose on the concentration of IgA and IgG_1_ (Table 3, Fig. 3); there were no significant effects of either Time p.i. or ln Dose on the concentration of IgG_2a_, IgG_2b_ and IgE (Fig. 3). The IgA response was the most immediate measurable response, being detectable on day 8 p.i., for the highest infective dose. This response also appeared to decline between days 15 and 22 p.i. for an infective dose of 750 (Fig. 3). This was in contrast to the dynamics of the IgG_1_, IgG_2a_ and IgG_2b_ responses which were delayed, comparatively, and which did not appear to have reached a maximum concentration. For IgE, the data suggest that there was a substantial response for the highest infective dose compared with all other doses (Fig. 3). The concentration of RMCP II in intestinal tissue was also significantly positively affected by both Time p.i. and ln Dose (Table 3, Fig. 3).

### 3.3 The host immune response and effects on *S. ratti* fitness

In order to identify which immune parameters affect *S. ratti* fitness, minimal models were determined by deletion testing from maximal models containing candidate explanatory variables: infective dose, time p.i. and immune parameters previously shown to be affected by *S. ratti* infection (i.e., IgA, IgG_1_, RMCP II, IL-4 and IL-13) (Tables 1–3). Many of these explanatory variables co-vary and thus the minimal models were used to determine which explanatory variables (i.e., dose, time p.i., individual immune parameters) exhibited the strongest association with survivorship and fecundity. Overall, this analysis showed that the density-dependent fitness of *S. ratti* was principally associated with the concentration of IgG_1_, IgA and IL-4 produced by MLN cells (Table 4).

*Strongyloides ratti* survivorship was significantly negatively associated with both the concentration of IgG_1_, IgA and IL-4 produced by MLN cells (Table 4). The IgG_1_ response had the greatest association with parasite survivorship, such that any effect of ln Dose on survivorship (e.g., Table 1) was no longer significant with the inclusion of IgG_1_. These IgG_1_, IgA and MLN IL-4 responses are, again, indicative of a Th2-type immune response.

For *S. ratti* fecundity, the model converged on that presented in Table 1, suggesting that there was no significant association with any immune parameters. However because, a priori, the host immune response is required for negative density-dependent effects on *S. ratti* fecundity (Paterson and Viney, 2002), we extended this analysis by using the actual estimated number of parasitic females present in each rat, ln Adult, rather than the treatment effect, ln Dose. Further, previously both ln Adult and ln Dose have explained similar and significant proportions of variance in *S. ratti* survivorship and fecundity (Paterson and Viney, 2002). This modified analysis revealed a significant negative association of IgA and *S. ratti* fecundity (Table 4), as well as significant associations of ln Adult and ln Adult * Time. Comparison of these two models of fecundity (Table 1 and 4) showed that the penalised log-likelihood score for the model that used ln Adult (415.9) was less than the model that used ln Dose (423.9), thereby suggesting that the former model was a better fit to the data.

Overall, both minimal models explaining parasite survivorship and fecundity (Table 4) show that these are negatively associated with a host Th2-type immune response, particularly the concentration of IgG_1_, IgA and IL-4 produced by MLN cells. Of these, IgG_1_ has by far the most significant negative association with parasite survivorship, but IgA is also negatively associated with both components of *S. ratti* fitness.

## 4 Discussion

The parasitic female stages of *S. ratti* experience a negative density-dependent reduction in fitness that requires the host immune response (Paterson and Viney, 2002). The majority of the measured components of the host immune response increased with the infective dose of *S. ratti*. Our statistical analyses indicated which of these measures exhibited the strongest association with parasite survivorship or parasite fecundity. The concentration of parasite-specific serum IgG_1_ and intestinal IgA showed the greatest negative association with *S. ratti* survivorship (Table 4) and the concentration of parasite-specific intestinal IgA showed the greatest negative association with *S. ratti* fecundity (Table 4). These results suggest that these antibodies may directly bring about these negative effects on these aspects of *S. ratti* fitness. Alternatively, it is also possible that these antibodies are intermediary factors in other host effector processes, or that they are correlated with other, unidentified, effector processes.

The apparent effect of IgG_1_ is consistent with observed effects of repeated different dose *S. ratti* infections on anti-*S. ratti* iL3 IgG titres (Uchikawa et al., 1991). However, IgA has not previously been implicated as part of the anti-*S. ratti* immune response of rats. Infections of both humans and dogs (Mansfield and Schad, 1992) with *S. stercoralis* results in an increase in the concentration of IgA. However, in dogs, there is no evidence that this has any functional effect against *S. stercoralis* (Mansfield and Schad, 1992). *Nippostrongylus brasiliensis* infections of rats also results in an increase in the concentration of intestinal parasite-specific IgA (Siński and Holmes, 1977).

For nematode infections, IgA has been shown to be associated with reductions in parasite fitness. In the gastrointestinal nematode parasite of sheep *Ostertagia circumcincta*, the concentration of anti-*O. circumcincta* larval IgA in serum was negatively correlated with worm length in lambs, which was itself inversely related with per capita fecundity (Stear et al., 1995, 2004; Davies et al., 2005). This, therefore, implies that the host IgA response results in smaller and less fecund *O. circumcincta*. A direct role for IgA in the control of *Trichinella britovi* infections in mice has also been made, since administration of an anti-*T. britovi* IgA mAb resulted in a dose-dependent reduction in establishment of larvae (Inaba et al., 2003).

To date there is little understanding of how specific antibody effector mechanisms could operate to bring about a reduction in parasite fitness. Antibody-containing plugs in the oesophagus of *S. ratti* have been observed (Moqbel and McLaren, 1980), which have been hypothesised to prevent feeding and thereby, presumably, to reduce survival and reproduction, and this may be how the effects of IgG_1_ and IgA on the fitness of *S. ratti* occur. Among the primary roles of IgG_1_ are opsonisation, the induction of phagocytosis and activation of the classical complement pathway. Complement has been implicated in the killing of pre-intestinal larvae of *S. stercoralis* (Ligas et al., 2003) and of *N. brasiliensis* in mice (Shin et al., 2001), which, presumably, will affect the rate of intestinal establishment of adult stages. Any such role against adult parasites established in the gut is not clear. There are a number of possible mechanisms by which IgA could affect parasite fitness, including by facilitating the transport of other effector molecules across the mucosal surface directly to the parasitic females (Kaetzel et al., 1991) or by activating eosinophils (Decot et al., 2005). Eosinophils have been repeatedly implicated in the expulsion of gastrointestinal nematodes (Onah and Nawa, 2000).

Low dose *S. ratti* infections resulted in a host immune response with characteristics of a Th1-type immune response, whereas for higher dose infections there was a Th2-type immune response (Fig. 2). This observation is therefore in agreement with previous analogous observations of other helminths (Bancroft et al., 1994; Dematteis et al., 2003; Babayan et al., 2005). Wild infections of helminth parasites are often likely to be at very low doses. For *S. ratti* in wild rats in the UK the mean intensity of infection is 37, the maximum is 173 (Viney, 2006). Combined, these laboratory and field studies suggest that the immune response of wild animals to helminths may not always be a Th2-type immune response.

Here, we observed a positive density-dependent effect on parasite fecundity (Table 1). This may be an artefact due to our over-estimation of fecundity (see Section 2.1) which may also differ across doses. However, this counter-intuitive observation is supported in part by a previous study in which fecundity increased slightly with density at the onset of patency but then decreased with density as the infection progressed (Paterson and Viney, 2002). The occurrence of positive density-dependent changes in parasite fecundity is also supported by the observation that in lines of *S. ratti* selected by the passage of larvae collected either early or late in an infection, their fecundity differed in their response to parasite density (Paterson and Barber, 2007). Such positive density-dependent effects may be advantageous because they enhance the transmission of individual worm genotypes in high-density infections before the onset of deleterious immune-dependent effects that, as we demonstrate here and earlier (Paterson and Viney, 2002), occur in high-density infections.

For *S. ratti*, time p.i. was the other major determinant of the change of the host immune response from a Th1- to a Th2-type profile, with animals exhibiting a progressively more Th2-type response through time, and the progression to a Th2-type response appearing to progress more quickly for higher dose infections (Fig. 2). Similarly, in endemic human *Ascaris lumbricoides* infections, the pronounced expression of cytokines and antibodies characteristic of a Th2-type immune response are only observed in older host age classes (Turner et al., 2003). In all, this may suggest that the low dose of helminth infections found naturally in the wild do not necessarily induce a Th2-type immune response, or do so only slowly, and that this non-induction of a Th2-type immune response may explain the likely chronicity of wild infections, compared with the relatively acute nature of laboratory infections (Kimura et al., 1999; Wilkes et al., 2004).

The immune-dependent density-dependent effects on components of fitness of *S. ratti* also occurs via immunological memory (Paterson and Viney, 2002). This observation may therefore suggest that the dose-dependent immune responses reported here also persist in immunological time. These observations have been made in hosts infected with a single species of pathogen. In the wild, different types (e.g., helminth, protozoa, bacteria, viruses) and species of infection are the norm and it can be envisaged that the immune responses induced by co-infecting species will quantitatively and qualitatively affect the effects reported here, though it is not yet possible to predict the nature of these effects.

Helminths are relatively large organisms (*S. ratti* is approximately 2 mm in length) and, a priori, even an infection with a single worm may be considered a large dose of antigen. As such, our observations of significant immunological effects of parasite dose across the range of six to 750 worms is, perhaps, surprising. This suggests that *S. ratti*, and perhaps other helminths, are functionally poor antigen sources either per se and/or as a consequence of their location within the host. A number of species of parasitic nematodes have been shown to regulate their hosts’ immune response, including suppression of specific portions of the response (Maizels et al., 2004) and such phenomena may be part of the density-dependent response observed here. However, there is no specific evidence for host immunomodulation by *S. ratti.*

Given that host immune responses are metabolically expensive, an individual should mount an immune response against a pathogen that is appropriate to the harm that is being caused (Medley, 2002). Thus, a cost-benefit analysis of the cost of infection and the cost of producing an immune response to ameliorate the infection, would generate an optimal immune response. For helminths, the amount of harm caused to a host is dependent upon the within-host burden of the helminth species in question. The immune-dependent, density-dependent effect on *S. ratti* fitness observed here is consistent with this prediction.

A key challenge for future research is to understand the immunodynamics of helminth, and other, infections; i.e., the quantitative relationships between parasite dose, the temporal nature and magnitude of the immune response and the effects of these immune responses on parasite fitness, and ultimately on the epidemiological dynamics of these infections. It is also crucial that these studies take careful note of the likely significant differences between laboratory and field settings.

## Figures and Tables

**Table 1:** Density-dependent effects on *Strongyloides ratti* infections, the effect of infective Dose and Time p.i. on survivorship and fecundity

	Term	Coefficient	*χ*^2^	*P*-value
Survivorship^a^	Constant	−6.69 ± 1.548		
	Time	0.74 ± 0.170	12.26	0.00046
	Time^2^	−0.022 ± 0.005	12.98	0.00031
	ln Dose	0.37 ± 0.228	2.59	0.107
	Time * ln Dose	−0.05 ± 0.016	7.27	0.0069
				
Fecundity^b^	Constant	6.76 ± 1.46		
	Time	−0.45 ± 0.20	3.96	0.046
	Time^2^	0.013 ± 0.006	3.22	0.07
	ln Dose	0.19 ± 0.088	4.05	0.044

a Response variable = number of parasitic females; offset variable = ln Dose; 2 × log-likelihood = −174.4, *k* = 4.39.

b Response variable = total viable egg output of parasitic females; offset variable, ln Adults; 2 × log-likelihood = −423.9, *k* = 1.32.

**Table 2:** The effect of infective Dose and Time p.i. on the concentration of IL-4 and IL-13 by mesenteric lymph node cells stimulated with parasitic female antigen

	Term	Coefficient	*χ*^2^	*P*-value
IL-4^a^	Constant	−5.52 ± 1.36		
	Time	0.265 ± 0.067	13.87	0.0034
	ln Dose	1.35 ± 0.18	43.37	<0.0001
				
IL-13^b^	Constant	2.08 ± 2.05		
	ln Dose	5.26 ± 0.486	60.15	<0.0001

All *P*-values are Bonferroni corrected.

a 2 × log-likelihood = 167.5, *χ*^2^ = 48.64, *P* < 0.0001.

b 2 × log-likelihood = −269.2, *χ*^2^ = 60.15, *P* < 0.0001.

**Table 3:** The effect of ln Dose and Time p.i. on the concentration of parasite-specific IgA in intestinal tissue, parasite-specific serum IgG_1_ and RMCP II in intestinal tissue

	Term	Coefficient	*χ*^2^	*P*-value
IgA^a^	Constant	−0.045 ± 0.013		
	Time	0.0013 ± 0.0004	10.72	0.0189
	ln Dose	0.005 ± 0.0014	22.21	<0.0001
				
IgG_1_^b^	Constant	−0.044 ± 0.0092		
	Time	0.0037 ± 0.0008	35.3	<0.0001
	ln Dose	0.0019 ± 0.0004	25.37	<0.0001
				
RMCP II^c^	Constant	−65.6 ± 16.74		
	Time	3.68 ± 0.825	17.38	0.0005
	ln Dose	10.01 ± 2.055	20.66	<0.0001

All *P*-values are Bonferroni corrected.

a 2 × log-likelihood = −66.03, *χ*^2^ = 28.6, *P* < 0.0001.

b 2 × log-likelihood = −104.8, *χ*^2^ = 48.48, *P* < 0.0001.

c 2 × log-likelihood = 281.3, *χ*^2^ = 32.92, *P* < 0.0001.

**Table 4:** The effects of component measures of the host immune response on the survivorship and fecundity of *Strongyloides ratti*

	Term	Coefficient	*χ*^2^	*P*-value
Survivorship^a^	Constant	−1.652 ± 0.25		
	IgG_1_	−0.17 ± 0.035	35.55	<0.0001
	IgA	−8.8 ± 2.1	11.72	0.0006
	IL-4 MLN	0.018 ± 0.005	11.07	0.0009
	IL-4 MLN^2^	−5.7 × 10^−5^ ± 2.3 × 10^−5^	5.54	0.0185
	Time	−0.08 ± 0.024	10.7	0.001
				
Fecundity^b^	Constant	3.43 ± 0.83		
	IgA	45.61 ± 15.3	6.11	0.013
	IgA^2^	−361.74 ± 140.5	4.3	0.038
	Time	0.018 ± 0.046	0.124	0.724
	ln Adults	1.38 ± 0.36	11.25	0.0008
	Time * ln Adults	−0.11 ± 0.02	15.07	0.0001

IgG_1_, mean = 3.13, range = 0–41.72; IgA, mean = 0.022, range = 0–0.12; IL-4, mean = 29.95, range = 0–211.7.

a Response variable = number of parasitic females; offset variable = ln Dose; 2 × log-likelihood = −151.9, *k* = 16.5.

b Response variable = total viable egg output of parasitic females; offset variable = ln Adults; 2 × log-likelihood = −415.9, *k* = 1.633.
